# District-level changes in low birth weight in India: National Family Health Surveys, 2016 and 2021

**DOI:** 10.1186/s12887-026-06913-4

**Published:** 2026-04-27

**Authors:** Mayanka Ambade, Shalem Balla, Sunil Rajpal, Rockli Kim, S. V. Subramanian

**Affiliations:** 1 School of Humanities and Social Sciences (SHSS), Indian Institute of Technology, Mandi, Himachal Pradesh India; 2 Department of Economics, FLAME University, Pune, India; 3 Division of Health Policy and Management, College of Health Science, Korea University, Seoul, South Korea; 4 Interdisciplinary Program in Precision Public Health, Department of Public Health Sciences, Graduate School of Korea University, Seoul, South Korea; 5 Harvard Center for Population and Development Studies, 9 Bow Street, Cambridge, MA 02138 USA; 6 Department of Social and Behavioral Sciences, Harvard T. H. Chan School of Public Health, Boston, MA USA

**Keywords:** Low birth weight, Districts, Geographic variation, India

## Abstract

**Background:**

Currently, concerns persist regarding the slow and inconsistent progress in reducing low birth weight children, especially in India, and little is known about the geographical variability of the trajectories of its change.

**Methods:**

This study analyses data from the fourth and fifth rounds of India’s National Family Health Survey (NFHS), conducted in 2015–2016 (NFHS-4) and 2019–2021 (NFHS-5), respectively. A four-level logistic regression model was used to calculate precision-weighted probabilities of live births reported as low birth weight at the cluster level; which were summarised by 720 districts.

**Results:**

As of 2021, around 18.2% of live births reported low birth weight in India, and there was no substantial improvement from 2016 to 2021. However, low birth weight declined by 2.05% points in the northern region and increased by 1.42% points in the eastern region. Out of 720 districts, 23.61% of districts experienced more than a 3% points reduction and 18.05% of districts experienced more than 3% points increase in low birth weight. The percentage of districts that have experienced more than 3% points increase varies from 28.57% in the eastern region to 8.51% in the southern region. While regional patterns of improvement and worsening are apparent, there are notable exceptions. For instance, districts such as Bangalore Rural in the south, Fatehabad in the north, Lohardaga in the east, and North Tripura in the northeast have shown improvements despite general trends of worsening in surrounding areas.

**Conclusions:**

Notable geographical variation in the percentage of low birth weight highlights the role of regional disparities in determinants of maternal and child healthcare. Addressing this issue necessitates an in-depth evaluation of policy implementation, incorporating best practices from districts that have demonstrated exceptions to the overall trends. These districts, which have successfully reduced the prevalence of low birth weight despite surrounding adverse conditions, are identified in this study.

**Supplementary Information:**

The online version contains supplementary material available at 10.1186/s12887-026-06913-4.

## Background

Low birth weight (LBW), defined as a newborn weighing less than 2500 grams, is a significant indicator of child health and early-life mortality [1]. Moreover, LBW has long-term implications for a child’s health, economic and educational outcomes, and developmental challenges [2, 3]. The long term consequences of malnutrition during fetal development and early infancy are well explained by Barker's hypothesis called ‘The fetal and infant origins of adult disease’, which posits that adverse conditions like malnutrition during fetal development and early infancy lead to long-term health conditions like cardiovascular diseases and metabolic diseases [4]. Thus, LBW serves as a proxy marker not only for neonatal vulnerability but also for adult health.

Globally, LBW accounts for 60–70% of neonatal deaths, underscoring its critical impact on child survival [1, 5]. Beyond individual health consequences, LBW has broader socioeconomic implications, including negative effects on human capital formation, productivity, and economic growth due to its long-term implications on health [6]. Recognizing these challenges, the World Health Assembly (WHA) in 2012 endorsed a Comprehensive Implementation Plan on Maternal, Infant, and Young Child Nutrition, which set forth six global nutrition targets, including a 30% reduction in the prevalence of LBW live births by 2025 [7]. Although LBW is not explicitly mentioned in the Sustainable Development Goals (SDGs), these targets would contribute to achieving SDGs related to nutrition (SDG-2), health (SDG-3), education (SDG-4), and economic growth by reducing inequalities (SDG-10) [8]. With the beginning of 2025, the time is contingent on studying changes in LBW to understand its temporal trajectory.

The aetiology of LBW is multifactorial, with existing studies identifying various contributing factors, such as genetic, nutritional, demographic, and psychosocial determinants, preterm birth, maternal morbidity, antenatal care, obstetric complications, and multiple pregnancies [9–17]. Currently, approximately one in seven children globally is born with LBW, with South Asia being home for 25% of children. Particularly in India, 27% of the children are born with LBW [1, 18]. Reducing LBW prevalence in India is essential for achieving global health goals. However, concerns persist regarding the slow and inconsistent progress in reducing LBW prevalence, especially in South Asia [1, 18]. Yet, empirical research, particularly in India, has largely focused on identifying the correlates of LBW, without adequately understanding if the changes in LBW are in desirable direction and pace, and how specific determinants, such as maternal dietary patterns and utilization of antenatal care services, which can be improved in the short term, can help to mitigate LBW prevalence [19–22].

India has implemented several policies and programs to address the multifactorial determinants of LBW. Initiatives such as the Janani Suraksha Yojana, Janani Shishu Suraksha Karyakram, and Integrated Child Development Services (ICDS) aim to enhance maternal nutrition, healthcare access, and the quality of antenatal care (ANC). For example, the ICDS provides supplementary nutrition and take-home rations for pregnant and lactating women, while ANC programs emphasize regular medical check-ups and iron-folic acid supplementation [23, 24]. Despite these interventions, significant gaps in program implementation and regional disparities in service coverage persist, particularly in areas with high LBW prevalence. Evaluating the effectiveness of these policies and identifying areas for improvement are essential for achieving sustainable reductions in LBW.

A critical knowledge gap exists in understanding whether regional variations in LBW trends reflect differences in the implementation of national policies and programs. This lack of understanding hinders a comprehensive assessment of how interventions have influenced LBW prevalence across India’s diverse geographic regions. District-level analysis is particularly crucial, as districts are the administrative units where policies are implemented, and variations in policy effectiveness are likely to manifest.

The availability of district-level data on LBW from the India’s National Family Health Survey (2016 and 2021) offers a unique opportunity to analyse changes in LBW prevalence over time. By examining these data, this study seeks to evaluate the progress of recent policy initiatives, identify regional disparities, and propose data-driven strategies for targeted interventions. This research aims to provide critical insights into the evolving landscape of LBW across India’s 720 districts, contributing to a deeper understanding of policy effectiveness and guiding future efforts to reduce LBW prevalence.

## Methods

### Data and sampling strategy

This study analyses data from the fourth and fifth rounds of India’s National Family Health Survey (NFHS), conducted in 2015–2016 (NFHS-4) and 2019–2021 (NFHS-5), respectively [25, 26]. For simplicity, in this study we use the end year of each survey for easy understanding. Both surveys are part of the Demographic and Health Surveys (DHS) program and collect extensive data on population health, nutrition, and well-being, including birth weight of the children. The survey design employed a stratified, multistage sampling approach, selecting clusters—defined as rural villages or urban wards—using probability proportional to size within districts of each state, followed by random selection of households within these clusters. Detailed documentation of the sampling strategy is available in the reports of the fifth round of NFHS [27].

### Outcomes

#### Low birth weight

According to the definition of the World Health Organization, LBW was defined as the child’s weight at birth being less than 2.5 kg (5.5 pounds) [28]. We analysed the percentage of live births reported LBW among children of 0–59 months across 720 districts in India, along with changes in absolute percentage points over time.

### District geometry

The clusters from NFHS-4 and NFHS-5 were redistributed to align with updated geographic boundaries, ensuring they matched accurately to 720 districts. For the majority of districts, the cluster-to-district linkages provided in the DHS microdata were preserved unchanged for all NFHS-5 clusters, except for those in Andhra Pradesh. Similarly, for NFHS-4 clusters, no adjustments were made where district boundaries remained consistent between NFHS-4 and NFHS-5. Consequently, the original cluster-to-district linkages were preserved for 694 districts in NFHS-5 and 564 districts in NFHS-4 without requiring adjustments. Further details are available elsewhere [29, 30].

### Statistical analysis

A four-level logistic regression model was used to calculate precision-weighted predicted probabilities of LBW among live births at the cluster level. The hierarchical structure includes individual i (level 1) nested within cluster j (level 2), which are nested within district k (level 3), and further nested within state l (level 4) as shown in (1):

$$Y_{ijkl}\;\mathit=\mathit\;\beta_{\mathit0}\mathit+\left(u_{jkl}\mathit+v_{kl}\mathit+f_l\right)$$

In the above-mentioned model, $\:{u}_{jkl}$, $\:{v}_{kl},\:{f}_{l}$ are model residuals specific to cluster, district, and state respectively. These residuals are assumed to have a normal distribution with a mean of 0 and a variance as shown in (2):

$$u_{jkl}\sim\left(0,\sigma_u^2\right);v_{kl}\sim\left(0,\sigma_v^2\right);f_l\sim\left(0,\sigma_f^2\right)$$

Here the term $\:{\sigma\:}_{u}^{2}$ denotes intra-district, and inter-cluster variation, $\:{\sigma\:}_{v}^{2}$ denotes within-state, and inter-district variation, and $\:{\sigma\:}_{f}^{2}$ stands for inter-state variation.

Using the STATA 18 and MLwiN 3.0 software program (using *runmlwin*) and the Monte Carlo Markov Chain (MCMC) method using the Gibbs sampler, multilevel modelling was performed, keeping the default prior distribution of Iterated Generalised Least Square (IGLS) as the starting value [31–34]. The district-level prevalence (in %) of each LBW is computed by taking the simple average of the cluster-level probabilities. This methodology was applied consistently across both NFHS-4 and NFHS-5 data to estimate the percentage of live births reported LBW for all 720 districts. Supplementary Fig. 5 provides the analytical framework of the study.

Changes in percentage from 2016 to 2021 were calculated as the percentage point difference by subtracting the 2016 prevalence from that of 2021. Geographic units were grouped into seven categories based on the direction and magnitude of change: less than − 5.00, -4.99 to -3.00, -2.99 to -1.00, -0.99 to 0.99, 1.00 to 2.99, 3.00 to 4.99, 5.00 and above. Further, for better geographical understanding, we have categorized all states and Union Territories into six regions as follows North: Jammu & Kashmir, Himachal Pradesh, Punjab, Chandigarh, Uttarakhand, Haryana, NCT Of Delhi, Rajasthan, Ladakh; North-East: Sikkim, Arunachal Pradesh, Nagaland, Manipur, Mizoram, Tripura, Meghalaya, Assam; South: Karnataka, Lakshadweep, Kerala, Tamil Nadu, Puducherry, Andaman & Nicobar Islands, Telangana, Andhra Pradesh; East: Bihar, West Bengal, Jharkhand, Odisha; West: Gujarat, Dadra & Nagar Haveli, Maharashtra, Goa; Central: Chhattisgarh, Madhya Pradesh, Uttar Pradesh [27].

In addition, districts identified as an Aspirational District Programme (ADP) by the government of India were examined separately. This programme was launched in 2018 by NITI Ayog as a policy initiative by the government of India aimed for accelerated development. Under this programme 117 districts were identified intentionally rather than post hoc classification bases on the progress made in 49 key performance indicators across 5 broad socio-economic themes: Health & Nutrition, Education, Agriculture & Water Resources, Financial Inclusion & Skill Development and Infrastructure [35]. ADP classification enables the assessment of these districts in comparison with other districts, as the districts targeted for accelerated development shows different trend. Of note, only 112 districts ultimately participated in the programme, as the state of West Bengal did not adopt it. Nevertheless, for the purposes of this analysis, we retained the original selection of 117 districts.

### Ethics statement

The Institutional Review Board of the International Institute for Population Studies (IIPS), Mumbai sanctioned data collection for both rounds of the NFHS encompassed in this study. This study does not meet the regulatory criteria for human subjects research as established by the Harvard Longwood campus, thereby exempting it from assessment.

### Role of the funding source

This research was funded by Bill & Melinda Gates Foundation, INV-002992. The funder had no involvement in any facet of this study, including study design, data analysis and interpretation, or manuscript writing.

## Results

### Sample characteristics

NFHS-4 contains unit-level records of 2,59,627 children and NFHS-5 contains unit-level records of 2,32,920 children less than 5 years of age. Due to the missing values, our analysis is restricted to unit records of 1,94,818 children in NFHS-4 (2016) and 2,09,222 children in NFHS-5 (2021) aged less than 5 years (Supplementary Table 1).

### All India trends in change of low birth weight children

**Fig. 1:**
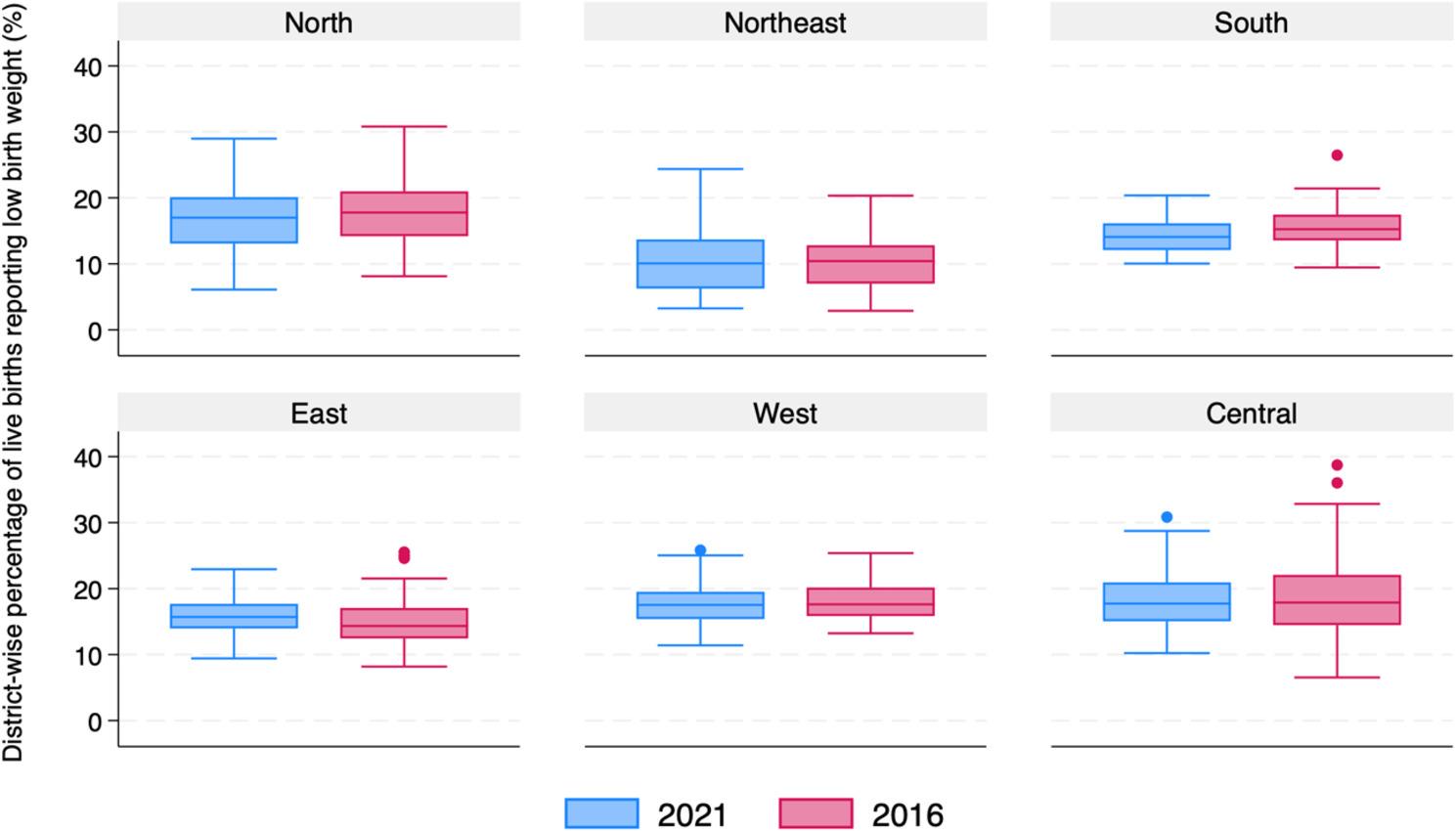
Box plots illustrating the percentage of live births reporting low birth weight in 2021 and 2016 by geographical regions

As of 2021, around 18.2% of live births reported LBW in India, and there was no change from 2016 to 2021. However, the change in LBW varies from -2.05% points (20.8% in 2016 to 18.7% in 2021) in the north region to 1.42% points in the east region (16.2% in 2016 to 17.6% in 2021) (Table 1; Supplementary Fig. 2; Fig. 1). In the Eastern region, the median prevalence across districts has increased from 14.3% in 2016 to 15.7% in 2021, and in the rest of the regions, the median percentage has remained the same (Fig. 1). In the aspirational districts, the median percentage has slightly increased from 14.9% in 2016 to 15.3% in 2021. Inter-district variation has changed across regions. Inter-quartile range (IQR) decreased from 6.02 in 2016 to 5.64 in 2021 in India. In geographical regions, in 2016 the central region had the highest IQR (7.61) and the southern region had the lowest (3.89). In 2021, the northeastern region had the highest IQR (7.44) and the eastern region had the lowest (3.72). IQR in the East, West and Central regions has decreased and the highest reduction was seen in the central region (1.73). IQR in the North, Northeast and Southern regions has increased and the highest increase was seen in the Northeastern region (1.64) (Fig. 1).

**Table 1:** Percentage of live births reporting low birth weight in India by geographical regions

Region	2021	2016	Change from 2016–2021
India	18.24	18.21	0.03
North	18.71	20.76	-2.05
North-East	14.93	14.69	0.24
South	16.04	16.65	-0.61
East	17.61	16.19	1.42
West	19.48	19.37	0.11
Central	19.88	20.09	-0.21

### Trajectories of change in percentage of LBW children across districts

Out of 720 districts in India, 23.61% districts have experienced more than a 3% point reduction, 19.44% have experienced a 1 to 2.99% point reduction, 20.14% have no change, 18.75% have experienced a 1 to 2.99% point increase, and 18.05% of districts have experienced more than 3% point increase in LBW from 2016 to 2021. The percentage of districts that have experienced more than a 3% point reduction varies from 38.97% in the northern region to 10.57% in the northeastern region. The percentage of districts that have experienced more than a 3% point increase varies from 28.57% in the eastern region to 8.51% in the southern region. In comparison with other districts, only 21.37% of aspirational districts experienced more than a 3% point reduction and 20.52% experienced more than a 3% point increase (Table 2).

**Table 2:** Number of districts by their improvement status for the percentage of live births reporting low birth weight in India by geographical regions, 2016-21

Region	less than -5.00	-4.99 to -3.00	-2.99 to -1.00	-0.99 to 0.99	1.00 to 2.99	3.00 to 4.99	5.00 and above	Total
India	12.78 (92)	10.83 (78)	19.44 (140)	20.14 (145)	18.75 (135)	9.44 (68)	8.61 (62)	100 (720)
Geographical Regions								
North	25.00 (34)	13.97 (19)	13.97 (19)	16.18 (22)	12.5 (17)	5.88 (8)	12.5 (17)	100 (136)
North-East	2.88 (3)	7.69 (8)	27.88 (29)	26.92 (28)	19.23 (20)	8.65 (9)	6.73 (7)	100 (104)
South	15.6 (22)	14.89 (21)	24.82 (35)	19.15 (27)	17.02 (24)	3.55 (5)	4.96 (7)	100 (141)
East	4.46 (5)	7.14 (8)	16.96 (19)	17.86 (20)	25 (28)	16.96 (19)	11.61 (13)	100 (112)
West	9.46 (7)	10.81 (8)	18.92 (14)	27.03 (20)	14.86 (11)	13.51 (10)	5.41 (4)	100 (74)
Central	13.73 (21)	9.15 (14)	15.69 (24)	18.30 (28)	22.88 (35)	11.11 (17)	9.15 (14)	100 (153)
Aspirational District								
Yes	12.82 (15)	8.55 (10)	15.38 (18)	24.79 (29)	17.95 (21)	10.26 (12)	10.26 (12)	100 (117)
No	12.77 (77)	11.28 (68)	20.23 (122)	19.24 (116)	18.91 (114)	9.29 (56)	8.29 (50)	100 (603)

The correlation between the percentage of LBW in 2016 and 2021 is positive and significant (*r* = 0.60; *p* < 0.01), indicating much improvement hasn’t been seen from 2016 to 2021. Most of the northeast region districts are concentrated in bottom-left-quadrant indicating that the percentage of LBW is low in both 2016 and 2021. Most of the central and eastern region districts are concentrated in the top-right-quadrant indicating that the percentage of LBW was high in both 2016 and 2021 (Supplementary Figs. 3 & 4).

**Fig. 2:**
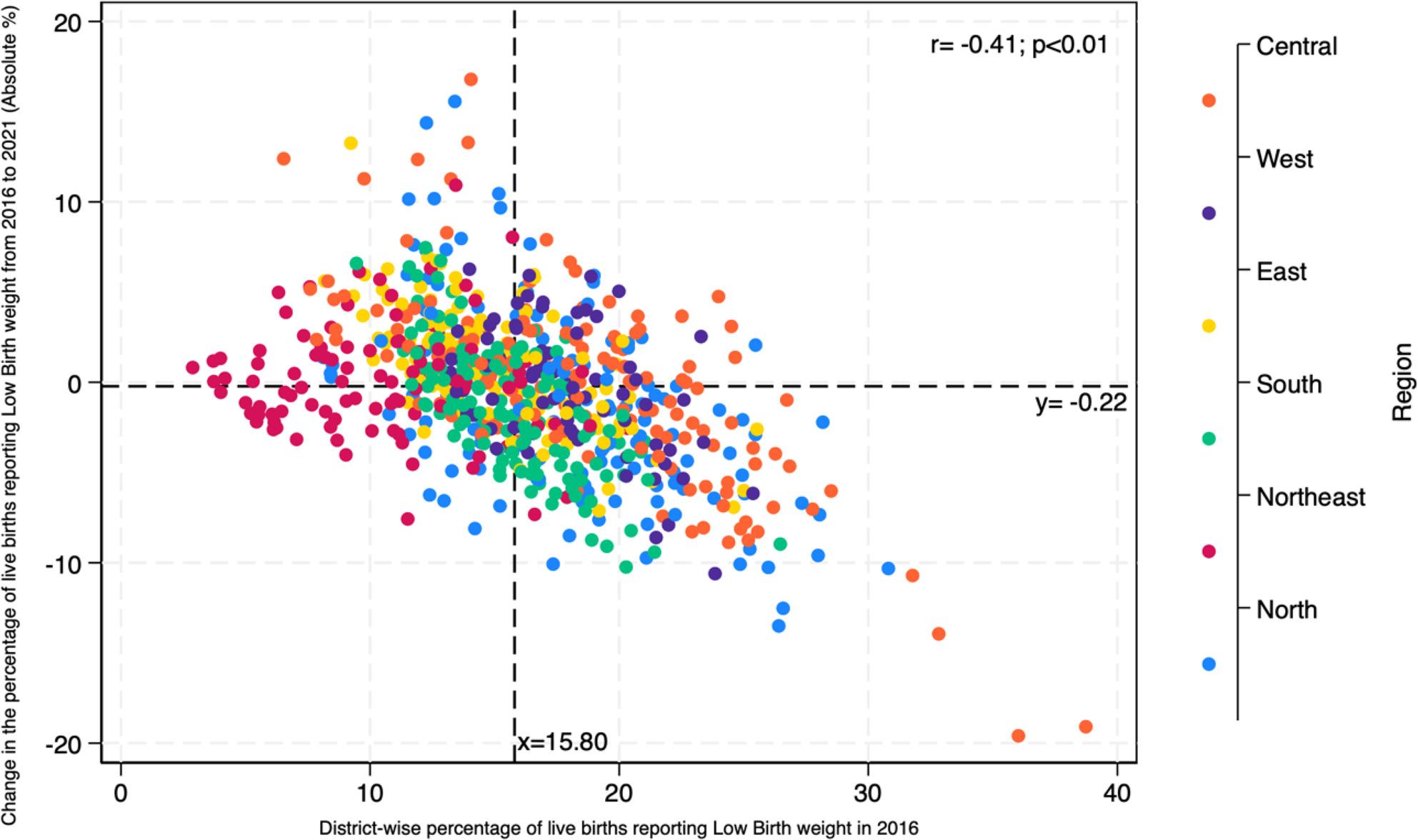
Scatter plot describing the correlation between the percentage of live births reporting low birth weight in 2016 and the absolute change from 2016 to 2021

In 2016 more than 20% of live births reported LBW in Punjab, Haryana, Gujarat, Madhya Pradesh, Maharashtra and some districts of Uttar Pradesh, Chhattisgarh, Odisha, and West Bengal. In almost all the districts in these states, the percentage remained the same and high in 2021 (Fig. 2 and Supplementary Figs. 1).

### Geographic variation in change of LBW children

There is a negative correlation between the percentage of live births reporting LBW in 2016 and the absolute change from 2016 to 2021 (*r* = -0.41, *p* < 0.01) suggesting districts with more LBW children have seen larger reduction from 2016 to 2021. There is no particular pattern observed by geographical regions. Many districts in the northeast region in the top-left quadrant suggest a relatively low percentage in 2016 and worsened from 2016 to 2021. The central region districts are concentrated in the bottom-right quadrant indicating districts with high percentages in 2016 have experienced a significant reduction from 2016 to 2021 (Fig. 2).

All districts in Sikkim, Arunachal Pradesh, Nagaland, Manipur, Mizoram, and Ladakh had low prevalence in 2016 and 2021, plus low headcount in 2021. Out of all the districts that had low prevalence in 2016, 62.78% of districts remained with low prevalence and low headcount in 2021. But, in 11% of districts both the prevalence and headcount increased in 2021. Out of all the districts that had a very high prevalence in 2016, 62.22% of districts remained with a high prevalence and high headcount in 2021. But, in 12.78% of districts both the prevalence and headcount decreased in 2021 (Supplementary Figs. 3).

### Contrasting trajectories in adjacent districts

**Fig. 3:**
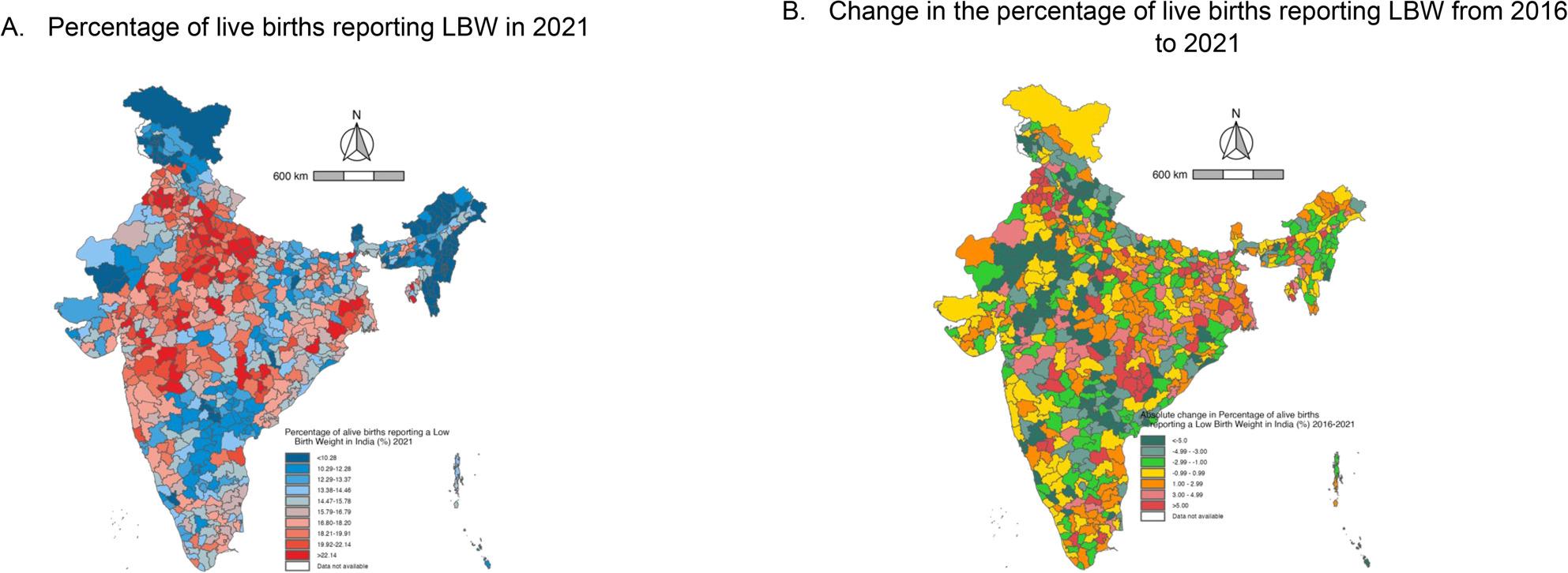
Maps of India illustrating the district-level percentage in 2021 and absolute change in the percentage of live births reporting low birth weight from 2016 to 2021

Even the neighbouring districts can experience different trajectories in LBW. For example, Y.S.R. Kadapa and Anantapuramu in Andhra Pradesh are neighbouring districts. But, Y.S.R. Kadapa has shown an increase in LBW of 5% point from 2016 to 2021 and Anantapuramu decreased by 3.7% point from 2016 to 2021. Belgaum in Karnataka showed a reduction of 5.7% point from 2016 to 2021. But, neighbouring districts Dharwad and Gadag have shown an increase of 5.8 and 7.5% points respectively. In Madhya Pradesh, the Sagar district has shown a rise of 11.3% point and the neighbouring district Vidisha has decreased by 6.8% point from 2016 to 2021 and districts such as Bangalore Rural in the south, Fatehabad in the north, Lohardaga in the east, and North Tripura in the northeast have shown improvements despite general trends of worsening in surrounding areas. These kinds of contrasting trajectories in adjacent districts are more common in the eastern and central regions (Fig 3).

## Discussion

Our study has three significant findings. First, strong regional gradients exist in temporal changes of LBW children. Districts in northern regions have experienced the greatest change between the two time periods after southern region, with a worsening of 30.9% of districts and an increase in the IQR by 0.25. Conversely, central districts have shown the biggest improvement, with an overall decline of 0.23% points and a reduction in the IQR by 1.73. Notably, northeastern districts have experienced the maximum increase in inter-district variability. Districts demonstrating significant improvements in reducing LBW prevalence are predominantly clustered in the southern region, while those with substantial worsening are concentrated in the eastern region. Second, the eastern and central districts currently bear the largest burden of LBW children in terms of headcount and prevalence. However, districts in the northern region exhibit the highest risk, characterized by high prevalence but lower headcount. These regions also display significant decreases in LBW prevalence over time. Third, while regional patterns of improvement and worsening are apparent, there are notable exceptions. For instance, districts such as Bangalore Rural in the south, Fatehabad in the north, Lohardaga in the east, and North Tripura in the northeast have shown improvements despite general trends of worsening in surrounding areas. These districts merit further investigation to understand the factors contributing to their positive outcomes. Such contrasting trajectories in adjacent districts are more common in the eastern and central regions.

Our findings should be interpreted with certain limitations. First, in case the mother-child card is unavailable, the observations on LBW are based on the mother’s recall. Given that some of these recalls could be as long as five years, there may be an issue of recall bias. Second, for districts with high non-institutional deliveries, many have not accurately reported/ measured birth weight leading to potential underestimation or overestimation. Third, the time between the two study years spans only three to five years which might not capture long-term trends nor the effects of policies and interventions. Fourth, COVID-19 pandemic which occurred between these two rounds of survey may have affected MCH outcomes through disruption in healthcare services and nutritional support systems. However given the cross-sectional nature of NFHS data, it is not possible to isolate and analyse the impact of pandemic on LBW. Lastly, estimates of districts of Andhra Pradesh may be underpowered as the geographical realignment created 26 districts from 13 districts. However, despite these limitations, the broader trends as visible in our study have been noted earlier [36–39], providing relevance to our more detailed findings.

Despite these limitations, our study offers comprehensive and critical insight and trends in LBW among newborns across districts in India. Our study underscores the significant regional variations in the improvement or worsening of LBW prevalence across the country. Notable geographical variation in LBW highlights the role of regional disparities in determinants of maternal and child healthcare [40–44]. Since LBW is primarily influenced by maternal factors such as the utilization of antenatal care (ANC) services during pregnancy, consumption of iron and folic acid supplements, adequate caloric intake, dietary diversity, and maternal health conditions like high blood pressure [45, 46], the impact of recent policy initiatives aimed at improving these factors warrants a robust assessment to explain the observed regional disparities in LBW outcomes. Of these factors, the role of ANC use is very significant. In fact, a mere single ANC visit during pregnancy reduces the risk of LBW by 54% [47]. Also, mothers with inadequate ANC are 16 times more likely to have a LBW child [48]. We observe that there is strong overlap in regions that have poor ANC coverage and worsening of LBW. For instance, nearly 52% of districts in the northern region have shown a deterioration in LBW prevalence, accompanied by increased inter-district variability. Notably, the northern districts also recorded the highest degree of worsening. Furthermore, empirical evidence suggests that while the uptake of ANC services has increased, the quality of these services, particularly for the recommended fourth or higher ANC visits in northern states remains suboptimal [49]. This issue is particularly pronounced in northern states such as Uttar Pradesh and Bihar, which together account for approximately 43% of the total headcount burden of poor-quality ANC services [50]. At the same time, central districts have seen improvements in LBW births, whereas states such as Madhya Pradesh, Uttar Pradesh, Chhattisgarh, and Jharkhand have seen a decline in inadequate ANC service use by more than 3.5% points, which is relatively higher than most states. Uttar Pradesh has seen a substantial decline of 6.6% points for inadequate ANC in the same time period.

Maternal factors such as weight and diet also play a critical role in determining LBW. In response, the Government of India has implemented several policy initiatives aimed at improving maternal nutrition. Programs such as the *Janani Suraksha Yojana* and *Janani Shishu Suraksha Karyakram* provide free rations to pregnant women, while the celebration of monthly Village Health, Sanitation, and Nutrition Days emphasizes nutritional requirements during pregnancy [51]. Additionally, the *Integrated Child Development Services* (ICDS) program offers free take-home rations to pregnant and lactating women. However, recent evaluations of the ICDS program highlight significant challenges. For example, the coverage of the Supplementary Nutrition Program (SNP), a component of ICDS, stood at only 37% for pregnant and lactating women in 2020, representing a decline of 11% points (approximately 2 crore women) from 2014. This decline coincides with an increase in LBW prevalence in central India from 2016 to 2021, despite improvements in headcount. Similarly, northeastern states, which have experienced the largest worsening in LBW prevalence, report poor ICDS coverage, compounded by issues with the quality and frequency of food distribution [52].

Beyond ANC utilization and maternal nutrition, other determinants of LBW, such as maternal age, literacy, poverty, and lifestyle factors like smoking and alcohol consumption, require long-term interventions. These factors fall outside the scope of this study, which focuses on changes in LBW prevalence over five years.

Finally, our study highlights exceptions to the general geographic patterns of LBW change, identifying districts that deviate from regional trends. The presence of such contrasting trajectories in adjacent districts in the eastern and central regions highlights the complexity of factors determining LBW. In districts like Belgaum in Karnataka where the percentage of LBW is high, the availability of health facilities continued to be very low compared to Dharwad and Gadag [53–55]. Even the maternal and other child health indicators are also poor in Belgaum compared to Dharwad and Gadag [55]. The study of districts that show exceptions to regional patterns helps us identify best practices too. For example, in Visakhapatnam, LBW prevalence has decreased slightly, despite increases in adjacent districts [52]. This improvement could be attributed to initiatives like the *Akshaya Patra Foundation*, which provides nutritious meals to all ICDS beneficiaries, including pregnant and lactating women. Similarly, Andhra Pradesh’s *Anna Amrutha Hastham* program aims to reduce LBW prevalence by offering complete meals through Anganwadi services. These examples underscore the importance of localized interventions in addressing LBW disparities.

## Conclusion

LBW, a critical determinant of child health and mortality, and a barrier to effective child growth and economic development, has worsened in nearly 37% of districts in India between 2016 and 2021. This underscore regional gradient, with substantial heterogeneity across neighbouring districts. Addressing LBW requires a district-sensitive approach that recognises local-level heterogeneity and leverages interventions at the local level. The observed pattern in district-level analysis aligns with frameworks emphasising spatial inequality and cumulative disadvantage. These districts largely overlap with those exhibiting poor-quality ANC coverage and inadequate implementation of ICDS programs. Addressing this issue necessitates an in-depth evaluation of policy implementation, incorporating best practices from districts that have demonstrated exceptions to the overall trends. These districts, which have successfully reduced LBW prevalence despite surrounding adverse conditions, are identified in this study.

## Supplementary Information

Supplementary Material 1.

## Data Availability

The dataset analysed during the current study are available in the DHS website: [https://dhsprogram.com/data/available-datasets.cfm](https:/dhsprogram.com/data/available-datasets.cfm) .
