# Supplementary material for: District-level changes in low birth weight in India: National Family Health Surveys, 2016 and 2021
Source: BMC Pediatr. 2026 Apr 27;26:448. doi: 10.1186/s12887-026-06913-4 (PMC13191964; doi:10.1186/s12887-026-06913-4)
Supplement: Supplementary file 1 — Supplementary Material 1. [file 12887_2026_6913_MOESM1_ESM.docx]

**Supplementary Text**

**Updating district Geographies and Shapefiles:**

The NFHS-2021 and NFHS-2016 surveys provide direct estimates for 707 districts and 640 districts, respectively, covering 28 States and 8 UTs. We updated the 707-district shapefile provided by the DHS Spatial Data Repository by dissolving ACs in Andhra Pradesh to 26 districts as per the CEO of Andhra Pradesh’s AC to District linkage details. Then, the external boundary of the district shapefile was updated to match the Survey of India’s external boundary for India.

NFHS-2016 and NFHS-2021 clusters were reassigned to the updated geographic boundaries for nesting within 720 districts. For districts, the cluster-to-district linkages that existed in the DHS microdata were taken as is without any adjustments made for all NFHS-2021 clusters except for the clusters that fell in the state of Andhra Pradesh and for all NFHS-2016 clusters where the district remained unchanged from NFHS-2016 to NFHS-2021. This resulted in 694 districts in NFHS-2021 and 564 districts in NFHS-2016 where no adjustments were made to the cluster-to-district linkages. For the clusters that fell into the remaining 26 districts in NFHS-2021 (all in Andhra Pradesh) and the 156 districts (76 parent districts) in NFHS-2016, a spatial join using ArcGIS Pro was used to assign clusters to the updated 720 district geometry. In this process, 54 clusters in NFHS-2016 were not assigned to any district because they did not fall into any eligible changed district that originated from their parent district. Therefore, the final number of clusters included in the district estimates was 30,170 (NFHS-2021) and 28,470 (NFHS-2016).

**Supplementary Table 1**: Study sample size selection from the two National Family Health Surveys, 2016–2021.

| **Wave** | **Sample size based on inclusion criteria (n)** | **Observations dropped due missing** | **Final Study Sample size (n)** |
| --- | --- | --- | --- |
| NFHS-5 (2019–21) | 2,32,920 | 23,698 | 2,09,222 |
| NFHS-4 (2015–16) | 2,59,627 | 64,809 | 1,94,818 |
| **All waves** | **4,92,547** | **88,507** | **4,04,040** |

**Supplementary Figure 1**: Maps of India illustrating the district-level percentage of live births reporting low birth weight in 2016


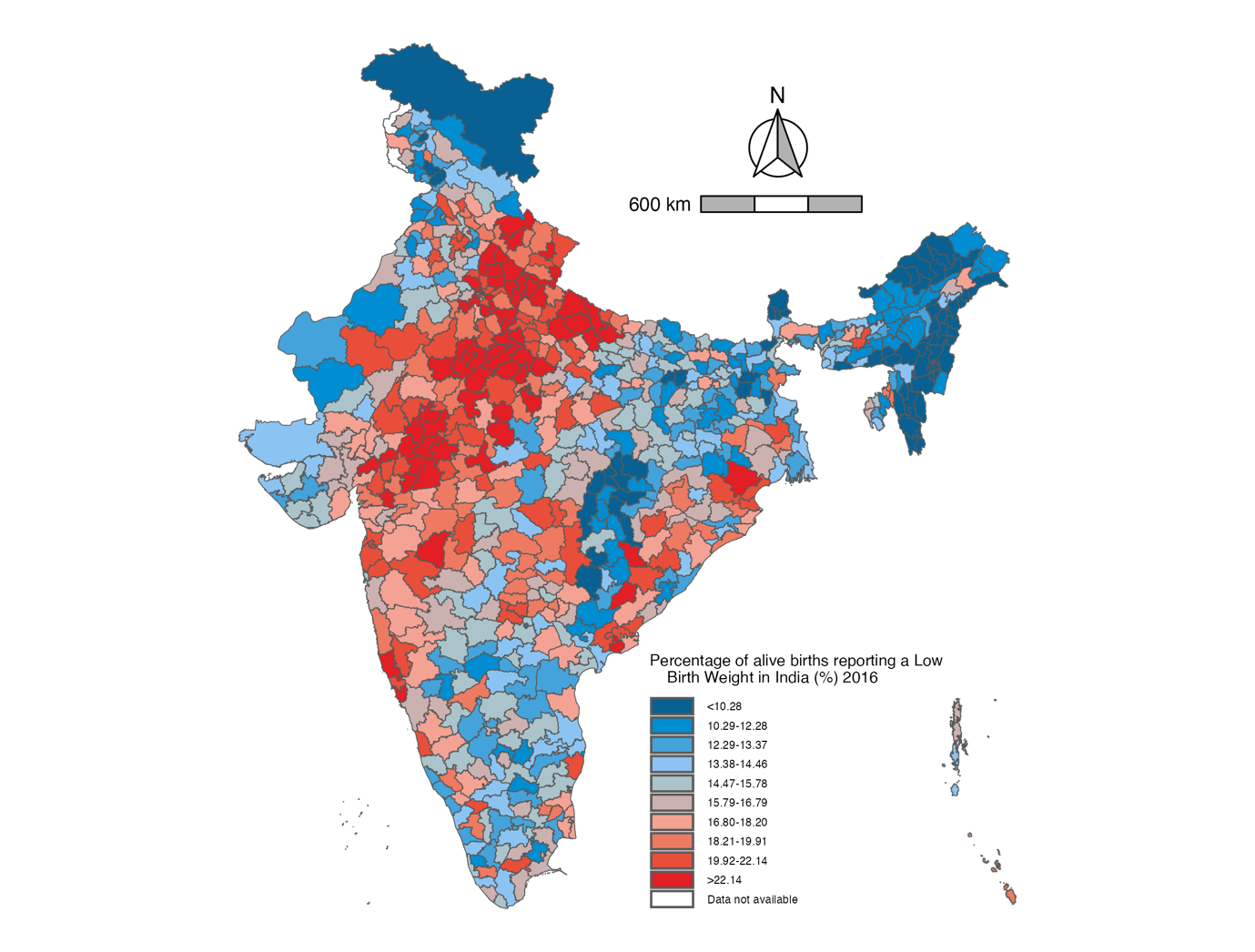


**Supplementary Figure 2**: Geographic variance partitioned between states, districts, and clusters for percentage of live births reporting low birth weight in 2016 and 2021

**Supplementary Figure 2**: Box plots illustrating the percentage of live births reporting low birth weight in 2021 and 2016

| 1. Overall | 1. By Status of District |
| --- | --- |
| 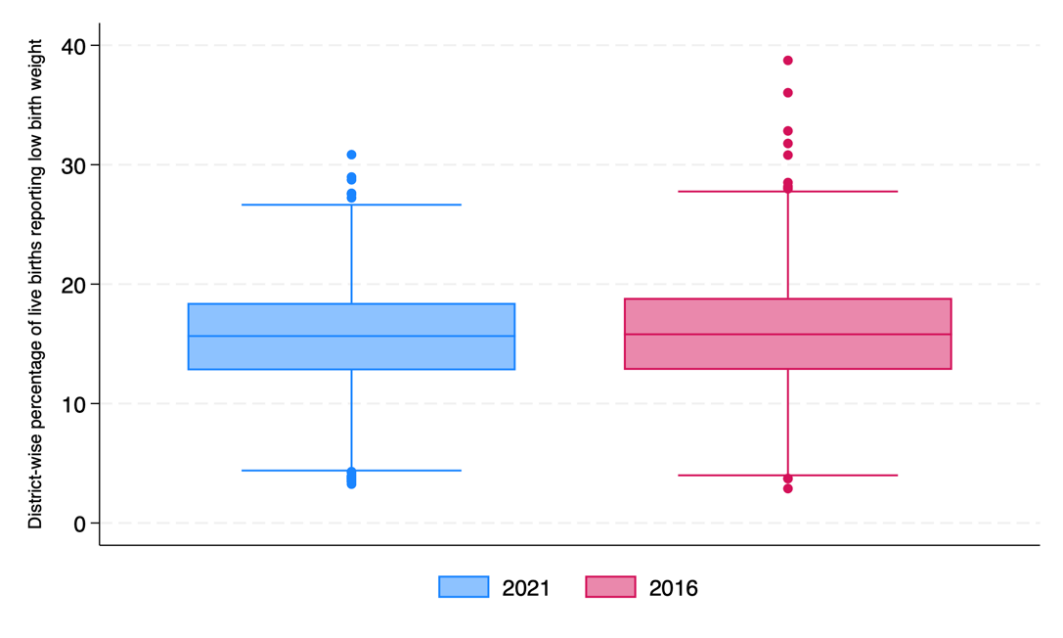 | 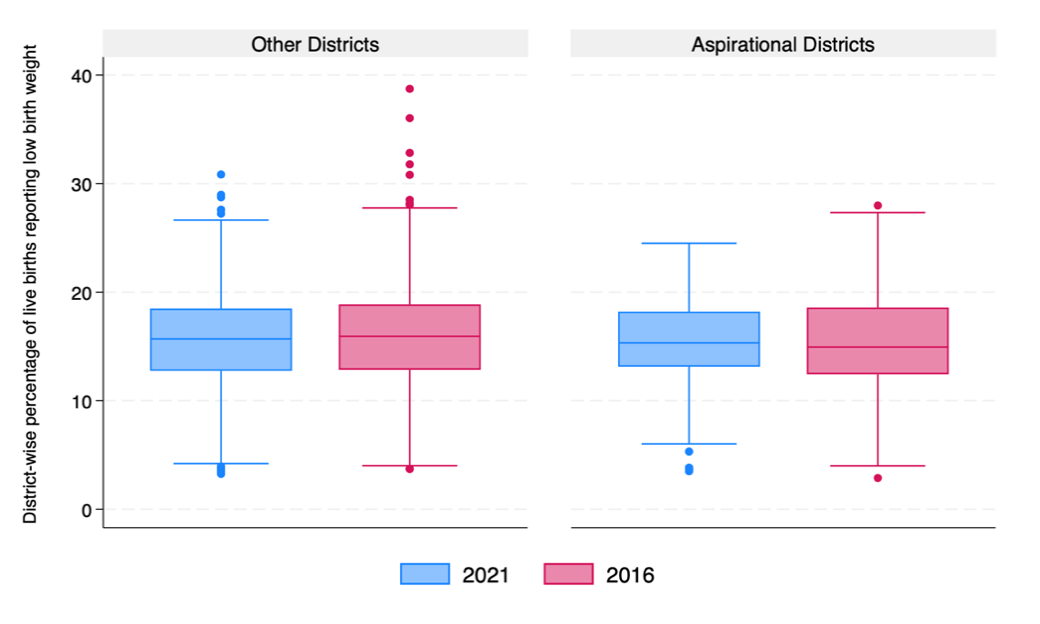 |

**Supplementary Figure 3:** Scatter plot describing the correlation between the percentage of live births reporting low birth weight in 2016 and 2021

| 1. Correlation between percentage in 2016 and 2021 | 1. Correlation between percentage in 2016 and relative change from 2016- 2021 |
| --- | --- |
| 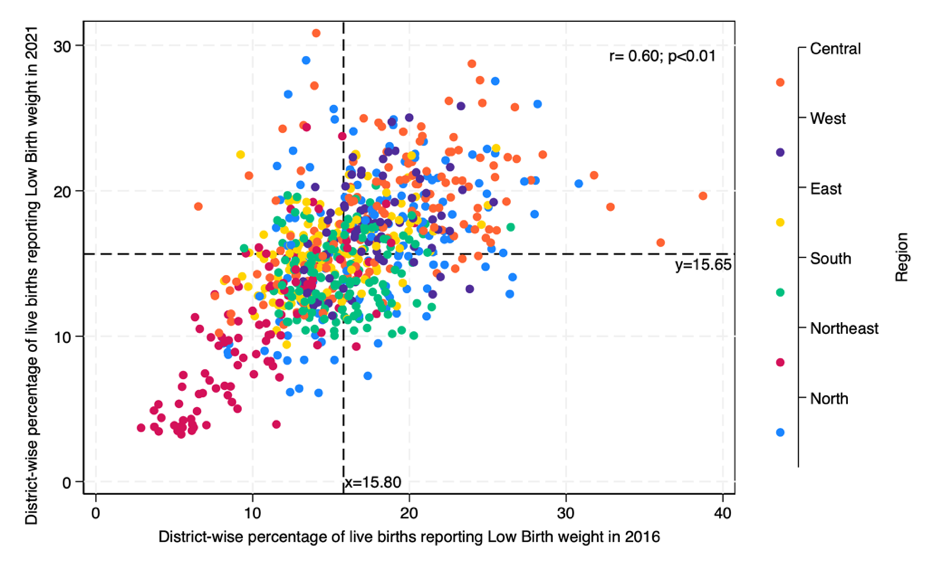 | 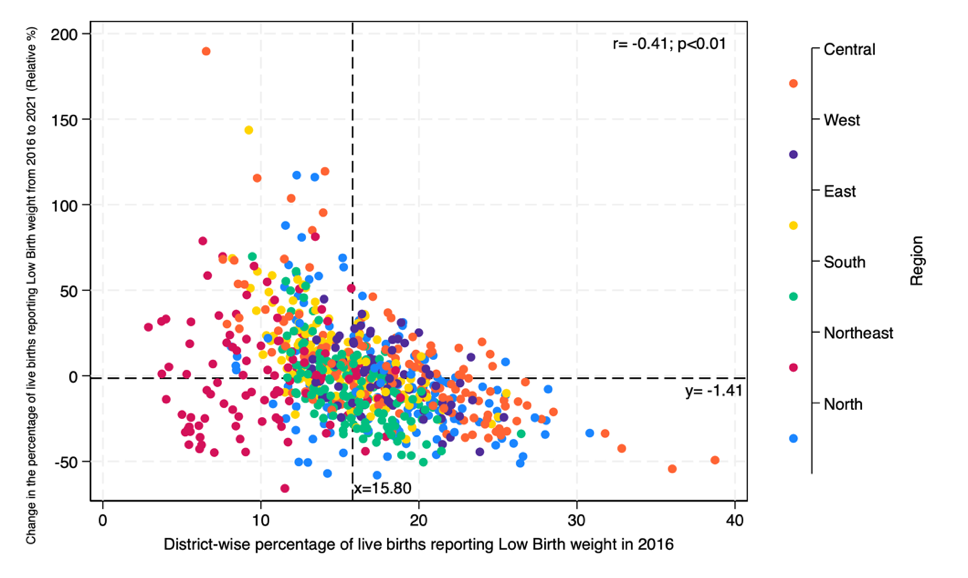 |
| 1. Correlation between percentage in 2021 and headcount coloured by percentage in 2016 | 1. Correlation between percentage in 2021 and headcount coloured by geographical region |
| 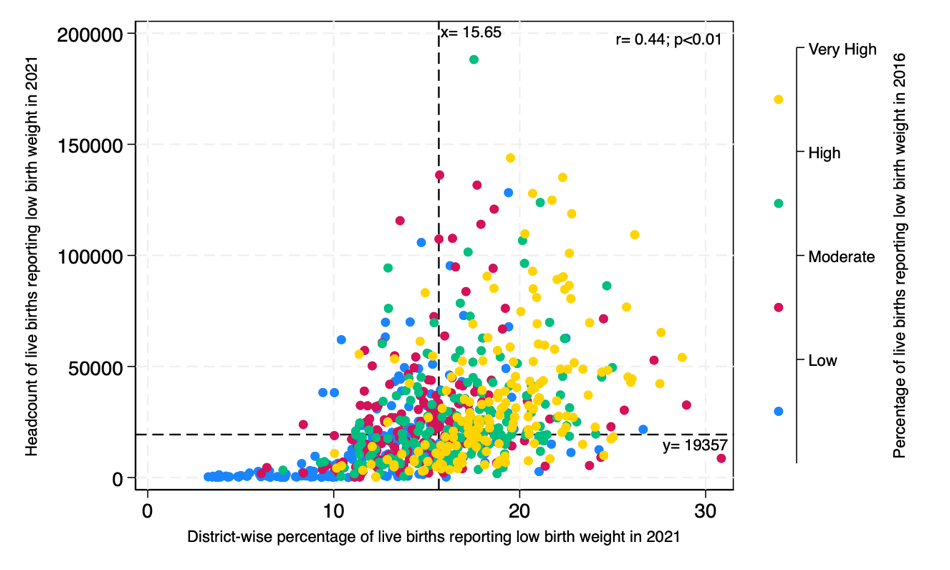 | 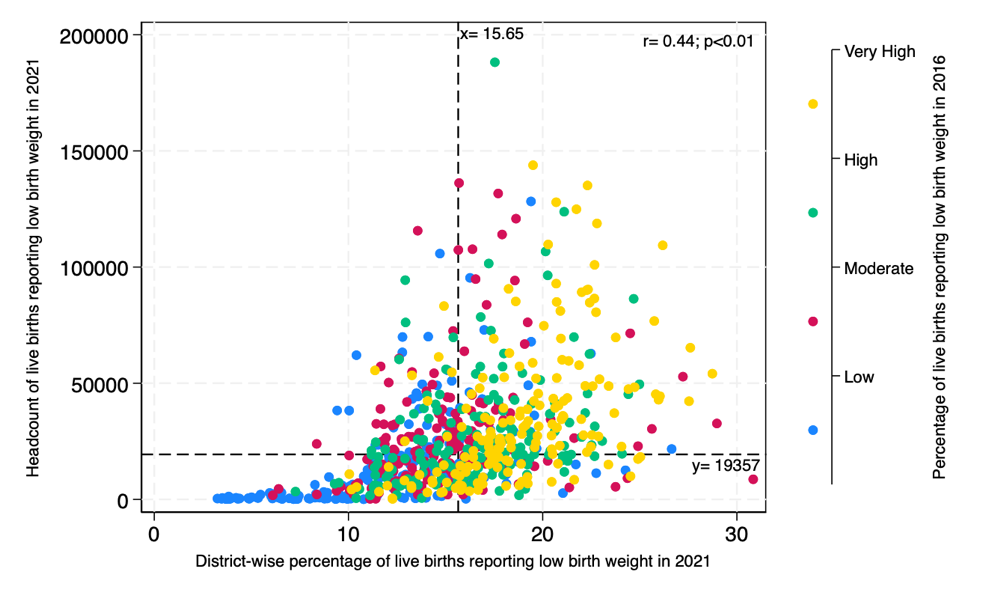 |

**Supplementary Figure 4:** Scatter plot describing the Spearman’s rank correlation between the rank of the district according to the percentage of live births reporting low birth weight in 2016 and 2021.


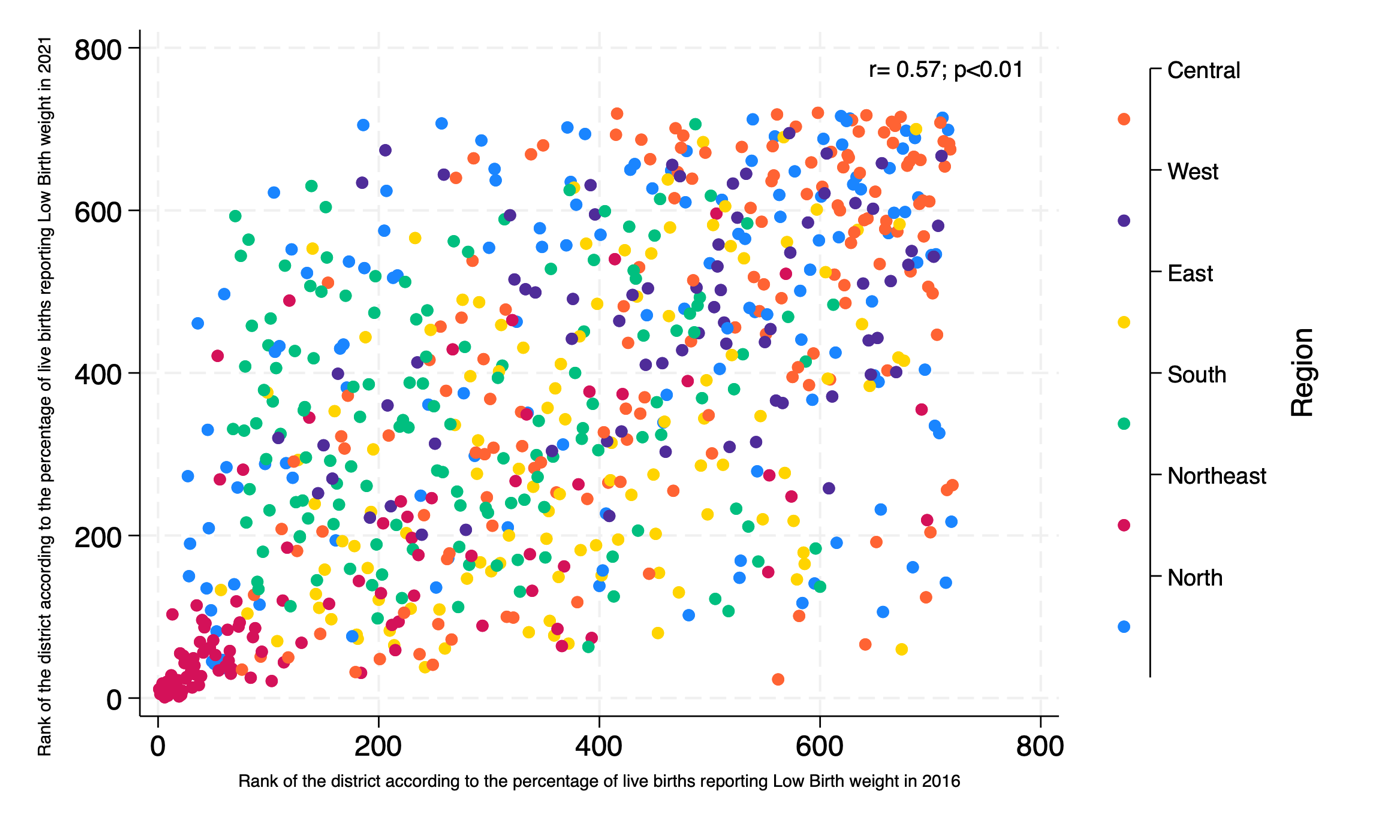


**Supplementary Figure 5:** Analytical framework and multilevel modelling approach
